# Kernel color and fertilization as factors of enhanced maize quality

**DOI:** 10.3389/fpls.2022.1027618

**Published:** 2022-11-21

**Authors:** Vesna Dragičević, Milan Brankov, Milovan Stoiljković, Miodrag Tolimir, Panagiotis Kanatas, Ilias Travlos, Milena Simić

**Affiliations:** ^1^ Group for Agro-ecology and Cropping Practices, Department for Breeding, Maize Research Institute “Zemun Polje”, Belgrade, Serbia; ^2^ Laboratory of Physical Chemistry, Vinča Institute of Nuclear Sciences, Belgrade, Serbia; ^3^ Department of Crop Science, University of Patras, Patras, Greece; ^4^ Laboratory of Agronomy, Agricultural University of Athens, Athens, Greece

**Keywords:** bio-fertilizer, organic fertilizer, kernel composition, essential elements, antioxidants, potential bio-availability, yield

## Abstract

Maize is an important staple crop and a significant source of various nutrients. We aimed to determine the macronutrients, antioxidants, and essential elements in maize genotypes (white, yellow, and red kernel) using three different fertilizers, which could be used as a basis to increase the nutrient density of maize. The fertilizer treatments used bio- and organic fertilizers as a sustainable approach, urea, as a commonly used mineral fertilizer, and the control (no fertilization). We evaluated the yield, concentration of macronutrient (protein, oil, and starch), nonenzymatic antioxidants (phenolics, yellow pigment, total glutathione (GSH), and phytic phosphorus), and reduction capacity of the 2,2-diphenyl-1-picrylhydrazyl (DPPH) radical, as well as essential elements that are commonly deficient in the diet (Mg, Ca, Fe, Mn, Zn, Cu, and S) and their relationships with phytic acid. The genotype expressed the strongest effect on the variability of grain yield and the analyzed grain constituents. The red-kernel hybrid showed the greatest accumulation of protein, oil, phenolics, and essential elements (Ca, Fe, Cu, and S) than a yellow and white hybrid, especially in the biofertilizer treatment. The yellow kernel had the highest concentrations of yellow pigment, GSH, phytic phosphorous, Mg, Mn, and Zn (19.61 µg g^−1^, 1,134 nmol g^−1^, 2.63 mg g^−1^, 1,963 µg g^−1^, 11.7 µg g^−1^, and 33.9 µg g^−1^, respectively). The white kernel had a greater starch concentration (2.5% higher than that in the red hybrid) and the potential bioavailability of essential metals, particularly under no fertilization. This supports the significance of white maize as a staple food in many traditional diets across the world. Urea was important for the enhancement of the antioxidant status (with 88.0% reduction capacity for the DPPH radical) and increased potential Zn bioavailability in the maize kernels (13.3% higher than that in the biofertilizer treatment). This study underlines the differences in the yield potential and chemical composition of red, yellow, and white-kernel maize and their importance as a necessary part of a sustainable human diet. This information can help determine the most appropriate genotype based on the antioxidants and/or essential elements targeted for kernel improvement.

## Introduction

Maize (*Zea mays* L.) is an important crop and a staple food worldwide. It is a source of many phytonutrients, mainly carbohydrates, as well as highly valuable proteins, oils, mineral nutrients, vitamins, secondary metabolites, phenolic compounds, and phytosterols. Zein, as the main protein in maize kernels, has significant applications in pharmacy and nutraceuticals, and resistant starch confers health benefits that may reduce the risk of some cancers, atherosclerosis, and metabolic syndrome (Sha et al., 2016). It is also important to emphasize that maize kernels are gluten-free and have a low glycemic index; thus, they could be included in various diets (Giuberti et al., 2015). While breeding was mainly focused on the yield potential rather than the chemical composition of the maize kernels, current trends in sustainable nutrition have improved the nutrient density of food. Because genotypes high in protein and antioxidants are low in yield (Mahan et al., 2013), maintaining a balance between yield potential and quality could become an important trait in the future.

Owing to the increased popularity of secondary metabolites and their antioxidant properties, genotypes with various kernel colorations, ranging from intense yellow to red, purple, or even blue and black, have received considerable attention (Žilić et al., 2012; Sha et al., 2016; Suriano et al., 2021); this explains the high antioxidant activity of maize flour than wheat flour (Nikolić et al., 2019). Nevertheless, in some regions, the white kernel is mainly used for human nutrition. The synthesis of antioxidants is primarily driven by environmental factors and genotype × environment interactions; thus, stressful conditions could increase the concentration of antioxidants, such as glutathione, phenolics, yellow pigment, and phytic acid (Phy), in the crop’s vegetative parts and grains (Brankovic et al., 2015; Dragičević et al., 2017; Saini and Keum, 2018).

Because a majority of soils are depleted and lack several essential elements, the deficiency of these nutrients (mainly Fe, Zn, I, and vitamin A) has resulted in the prevalence of “hidden hunger” worldwide. Addressing the need for nutrient-dense food requires strategies that improve food quality and benefit developing and developed countries (Lowe, 2021; FAO, 2022). Soils should receive significant attention as the nutrients in food originate here and as a resource with a limited or depleted nutrient budget. Therefore, the production system should integrate different practices, such as biofortification, which affect the mineral balance of the plants (Sofo et al., 2016). Fertilizers, such as organic fertilizers that are rich in various nutrients (in highly or less accessible forms) and biofertilizers, can enrich the soil with organic matter, promote soil microbiota, restore soil fertility, and increase crop fitness and growth. Roychowdhury et al. (2017) and Mishra et al. (2012) considered biofertilizers to be one of the best modern tools as an alternative to mineral fertilizers, with a beneficial impact on the environment and fulfilling the optimal supply of nutrients (P, Ca, Cu, and Zn) to the crops. Crop–microbiota relations are dynamic and depend on various factors. The diversity and number of rhizosphere microbiota are highly dependent on the crop phenophase and the type and amount of fertilizer used (Vollú et al., 2018). Furthermore, nutrient absorption and remobilization are highly dependent on the genotype (Ray et al., 2020), which should also be considered when nutrient-dense yields are the goal.

Although grain enrichment with essential elements has been targeted in previous studies, the factors that promote or reduce their bioavailability in the digestive organs of humans and monogastric animals must also be considered. In this context, phytic acid is the chief antinutrient. It is primarily a phosphorus reserve in seeds and grains, with the ability to bind minerals, proteins, and starch, limiting their bioavailability. However, its benefits are reflected in its high antioxidant activity, preventing lipid peroxidation and, thus, preserving food by preventing it from changing color and spoiling (Feizollahi et al., 2021). It can also reduce the risk of certain cancers, support heart health, and manage renal stones. It was ascertained that an increase in the phytic acid concentration in plants is also related to climate change, i.e., the atmospheric CO_2_ rise, which additionally decreases the accessibility of mineral elements (Chaturvedi et al., 2017; Perera et al., 2018). Therefore, to increase the bioavailability of essential elements, it is important to determine their distribution in grains and seeds and their ratio with phytic acid (Johnson et al., 2013; Wang et al., 2015). For example, P, K, Ca, and Fe are mainly present in the rice aleurone; Zn is distributed from the aleurone to the inner endosperm, and Cu is mainly located in the inner endosperm and is not associated with P (Iwai et al., 2012).

The nutritional quality of the maize grain, especially for genotypes with different kernel colors, was narrowly described, with no data regarding essential elements and factors that promote/reduce their potential bioavailability. Consequently, this study aimed to determine the macronutrients, antioxidants, and essential elements in three different maize genotypes (white, yellow, and red) under the influence of three types of fertilizer treatments, which could be used as a basis for increasing the nutrient density of maize. The fertilization practices included sustainable fertilizers, bio- and organic fertilizers, and urea, as a commonly used mineral fertilizer. The effects of the treatments were determined by evaluating yield, macronutrients (protein, oil, and starch), various nonenzymatic antioxidants (phenolics, yellow pigment, total glutathione, phytic phosphorus, and the reduction capacity of the 1,1-diphenyl-2-picrylhydrazyl (DPPH) radical), and essential elements that are commonly deficient (Mg, Ca, Fe, Mn, Zn, Cu, and S) and their relations with phytic acid, including the potential bioavailability.

## Material and methods

### Trial settings and soil properties

The experiment was conducted during the maize vegetative period in 2018−2020 under dry farming conditions in Zemun Polje, Serbia (44° 52′ N, 20° 20′ E). The soil was slightly calcareous chernozem, containing 53.0% sand, 30.0% silt, and 17.0% clay. The preceding crop was always winter wheat (*Triticum vulgare* L.). Each year, at the beginning of April, the soil was sampled, and the chemical composition, including the pH, soil organic matter content (SOM), and contents of the available elements, were determined (**Table 1**). The variations in the contents of the mineral elements were slight and could be owing to the soil conditions of the experimental area, the previous crop, and/or the meteorological conditions and variability in the growing conditions.

**Table 1:** The soil composition, including soil organic matter (SOM) content and available forms of mineral elements.

	N	pH	SOM	P	K	Mg	Ca	Fe	Mn	Zn	Cu	S
	kg ha^−1^		%	kg ha^−1^		mg kg^−1^						
Depth (cm)	0–90	0–30										
2018	166.4	7.17	2.82	57.9	158.2	385.2	736.4	21.98	23.5	3.86	3.31	451.54
2019	153.9	7.19	2.88	60.5	160.2	394.75	695.0	18.83	17.4	4.32	4.65	448.49
2020	167.5	7.16	3.13	61.2	162.1	342.08	732.39	24.64	18.54	5.42	4.61	448.87

The experimental trial included maize hybrids with different kernel colors: red (ZP 5048*c*), white (ZP 522*b*), and intense yellow (ZP 737). The sowing was performed in the spring (last week of April) in all 3 years. Together with seed-bed preparation (2–3 days before sowing), the fertilizer treatments were applied and incorporated into the soil according to the manufacturer’s recommendations: biofertilizer (BF; Team Micorriza Plus), 3 kg ha^−1^ (0.5 kg 100 L^−1^ water); organic fertilizer (OF; Fertor), 2.5 t ha^−1^; mineral fertilizer (urea; 46% N), 200 kg ha^−1^; and control (Con; no fertilization). Team Micorriza Plus is an inoculum in powder form that contains the arbuscular mycorrhizal fungi *Rhizophagus intraradices* (150 spores g^−1^) and *Glomus mosseae* (150 spores g^−1^) and rhizosphere bacteria (1 × 10^7^ UFC g^−1^) with 56% organic matter content. It improves plant nutrient absorption, increases the crop’s tolerance to abiotic and biotic stresses, and maintains soil fertility. Fertor is a fertilizer in pellet form, produced from chicken manure and plant-based organic matter. It contains NPK (4.5:2.7:2.3) + 1.1% Mg + 9.3% Ca and other macro- and micronutrients, which are partly soluble and available to plants, whereas insoluble parts enable the continual release of nutrients during vegetation. Standard cropping practices were applied, according to the manufacturer’s requirements. Both fertilizers, Team Micorriza Plus and Fertor, are permitted for use in organic agriculture. After harvest (during the second half of October), the maize grain yield was measured and calculated at 14% moisture, and the grains were used for further analyses.

### Chemical analyses

The concentrations of protein, oil, and starch in the maize kernels were determined using a near-infrared analyzer (Infraneo, Chopin, France) and were presented as a percentage. The kernel samples (100 g) were milled on the Perten 120 (Perten, Stockholm, Sweden; particle size < 500 μm). The antioxidants, such as phytic phosphorus (Pphy) and total glutathione (GSH), were determined after extraction with 5% trichloroacetic acid. The extract was centrifuged at 12,000 rpm for 15 min (Model Velocity 18R Versatile Centrifuge, Rotor TA15-24-2; Dynamica Scientific, Livingston, UK) at 4°C, and the absorbance was measured using a spectrophotometer (Biochrom Libra S22 UV/Vis Spectrophotometer, Biochrom, Cambridge, UK). The Pphy concentration was determined using the method described by Dragičević et al. (2011), which is based on the pink color formed upon the reaction between ferric ion and sulfosalicylic acid from the Wade reagent; the absorbance was measured at *λ* = 500 nm. The GSH was determined using the method proposed by Sari-Gorla et al. (1993), by adding 0.2 M potassium phosphate buffer (pH = 8.0) and 10 mM 5.5′-dithio(2-nitrobenzoic acid) to the extract and measuring the absorbance at 415 nm.

Water-soluble phenolics were determined after extraction with double-distilled water and centrifugation at 12,000 rpm for 15 min, using the method proposed by Simić et al. (2004). After adding 0.05M FeCl_3_ in 0.1 M HCl and 0.008 M K_3_Fe(CN)_6_ to the sample solution, the absorbance was measured at *λ* = 722 nm, and the concentration of phenolics was expressed in micrograms of ferulic acid equivalent. The yellow pigment (YP) was determined using the method proposed by Vancetovic et al. (2014) after extraction with 1-butanol and centrifugation at 10,000 rpm for 5 min; the absorbance was measured at *λ* = 436 nm and expressed in micrograms of β-carotene per gram.

The scavenging activity, i.e., the reduction capacity of free radicals, was determined using the method suggested by Abe et al. (1998). After extraction with 70% acetone, the difference between the blank and the sample containing the added DPPH radical was measured, and the reduction capacity was displayed as the percentage of DPPH reduction capacity. The concentrations of the essential elements Mg, Ca, Fe, Mn, Zn, Cu, and S were determined after wet digestion with an HClO_4_ + HNO_3_ mixture, using inductively coupled plasma-optical emission spectrometry (Spectroflame, 27.12 MHz and 2.5 kW, model P, Spectro Analytical Instruments, Kleve, Germany).

### Meteorological conditions

Each experimental season in 2018–2020 was characterized by an optimal total precipitation amount (**Table 2**), ranging from 327.7 (2020) to 366.0 mm (2019). However, the distribution was unequal, with lower values in April and September of 2018 and 2020 (the minimum value was achieved in April 2020, with only 4.7 mm precipitation). Similarly, a low precipitation amount occurred in August−September 2019. With respect to temperature fluctuations, 2018 had the highest temperature on average. The highest values mainly occurred in August in all 3 years, and the highest value was 25.9°C in 2019.

**Table 2:** The mean temperature (°C) and precipitation sum (mm) at Zemun Polje during the maize growing period in 2018–2020.

Months	Average temperature (°C)			Precipitation sum (mm)		
	2018	2019	2020	2018	2019	2020
April	18.0	14.6	14.4	24.6	51.3	4.7
May	21.7	15.7	16.9	39.0	129.6	79.9
June	22.7	24.2	21.3	150.1	113.7	125.9
July	23.6	24.1	23.3	61.9	31.0	34.8
August	25.7	25.9	25.2	44.0	19.8	66.3
September	19.8	18.6	21.9	16.9	20.6	16.1
Average/sum	21.9	20.5	20.5	336.5	366	327.7

### Statistical analyses

The data were processed using an analysis of variance (*F*-test), with a significance level of *p* < 0.05. Moreover, a correlation analysis (Pearson’s coefficients) included the correlation between the GY and analyzed elements (Mg, Ca, Fe, Mn, Zn, Cu, and S) and between the DPPH reduction capacity and concentration of the analyzed antioxidants (Pphy, GSH, phenolics, and YP), at *p* < 0.05. The results for the element removal with yield and the relation between Phy and the essential metals were presented as a mean ± standard error. Furthermore, the interdependence between the applied treatments and genotypes with respect to the kernel chemical composition was analyzed using a principal component analysis (PCA) as a dimensionality-reduction method, and the analysis was performed using SPSS for Windows Version 15.0 (SPSS, 2006).

## Results

### Impact of the year, fertilization treatments, and kernel color on the variation in the yield and chemical composition of the maize kernels

The sources, such as the year, fertilizer, genotype, and their interaction, exhibited a significant impact on the variability of the tested parameters (**Table 3**). In 2018, the highest average levels of GY and oil concentration were recorded (17.5% and 0.17%, respectively, greater compared to the levels in 2020), as well as Pphy, DPPH reduction capacity, GSH, Mn, and Zn (5.8%, 4.1%, 7.4%, 10.0%, and 27.6%, respectively, higher compared to the levels in 2019); protein, phenolics, YP, Ca, and Cu had the greatest values in 2020 (to 0.74%, 12.2%, 33.8%, 57.5%, and 55.9%, respectively, greater compared to the values in2018), as well as Fe and S (to 20.9% and 12.3%, respectively, compared to values in 2020). With regard to the fertilizer treatments, the highest average values for GY, Fe, and Zn were achieved in the BF treatment (2.4%, 40.0%, and 12.9% respectively, greater than those for the control); the greatest accumulation of protein, oil, Mg, Ca, Mn, Cu, and S occurred in the OF treatment (to 0.38%, 0.17%, 8.5%, 25.6%, 12.8%, 31.7%, and 12.4% respectively, greater than control); and the highest values for Pphy, phenolics, GSH, and DPPH were in the urea treatment (to 1.8%, 8.0%, 23.4%, and 0.5%, respectively, greater than for control). The control only showed an increase in the average starch and YP values.

**Table 3:** The analysis of variance includes the effect of the year (Y), fertilizer treatment (F), genotype (G), and their interaction on the grain yield (GY), protein, oil, starch, phytic phosphorus (Pphy), phenolics (Phen), yellow pigment (YP), and glutathione (GSH) contents, reduction capacity of the DPPH radical (DPPH), and concentrations of Mg, Ca, Fe, Mn, Zn, Cu, and S in maize grains with different kernel colors.

SOF	*df*	GY	Protein	Oil	Starch	Pphy	Phen.		YP	GSH	DPPH		Mg	Ca	Fe	Mn	Zn	Cu	S
Repl.	4	F																	
Y	2	464.4^*^	27.94^*^	3.17^*^	0.55	42.47^*^	7.07^*^		17.32^*^	0.74^*^	10.53^*^		26.66^*^	101.85^*^	6.56^*^	6.40^*^	78.15^*^	159.44^*^	68.08^*^
G	2	0.95^*^	28.22^*^	312.54^*^	30.62^*^	8.49^*^	87.49^*^		84.46^*^	2.24^*^	48.69^*^		20.16^*^	0.82	9.92^*^	22.85^*^	2.98^*^	0.01	3.68^*^
F	3	0.05	2.03^*^	0.36	1.09^*^	0.30	0.17		0.04	2.07^*^	0.16		3.89^*^	2.87^*^	24.18^*^	7.08^*^	5.01^*^	3.06^*^	4.24^*^
Y × G	35	210.74^*^	134.15^*^	176.09^*^	45.38^*^	37.93^*^	89.91^*^		792.53^*^	2.35^*^	55.48^*^		55.16^*^	29.81^*^	7.32^*^	34.75^*^	47.35^*^	51.01^*^	30.08^*^
Y × F	47	64.85^*^	6.80^*^	0.71	0.57	8.58^*^	1.60^*^		2.92^*^	1.74^*^	2.07^*^		7.56^*^	43.19^*^	13.85^*^	3.80^*^	24.09^*^	77.92^*^	37.59^*^
G × F	47	64.82^*^	7.93^*^	67.9^*^	6.99^*^	2.06^*^	16.49^*^		14.25^*^	2.08^*^	10.46^*^		5.83^*^	1.13^*^	12.12^*^	8.34^*^	2.04^*^	0.88^*^	2.04^*^
Y × G × F	143	47.99^*^	21.09^*^	81.34^*^	19.20^*^	30.43^*^	101.11^*^		338.52^*^	3.12^*^	224.87^*^		321.12^*^	1,201.6^*^	612.22^*^	131.04^*^	420.65^*^	863.88^*^	1,939.1^*^
*p*_0.05_																			
Y		0.000	0.000	0.046	0.578	0.000		0.001	0.000	0.478		0.000	0.000	0.000	0.000	0.002	0.000	0.000	0.000
G		0.395	0.000	0.000	0.000	0.000		0.000	0.000	0.111		0.000	0.000	0.443	0.000	0.000	0.055	0.986	0.028
F		0.995	0.114	0.779	0.355	0.803		0.919	0.991	0.108		0.925	0.011	0.040	0.000	0.000	0.003	0.032	0.007
Y × G		0.000	0.000	0.000	0.000	0.000		0.000	0.000	0.023		0.000	0.000	0.000	0.000	0.000	0.000	0.000	0.000
Y × F		0.000	0.000	0.727	0.852	0.000		0.110	0.002	0.076		0.030	0.000	0.000	0.000	0.000	0.000	0.000	0.000
G × F		0.000	0.000	0.000	0.000	0.031		0.000	0.000	0.029		0.000	0.000	0.345	0.000	0.000	0.033	0.560	0.033
Y × G × F		0.000	0.000	0.000	0.000	0.000		0.000	0.000	0.000		0.000	0.000	0.000	0.000	0.000	0.000	0.000	0.000
CV (%)		3.08	3.42	4.57	2.42	4.51		5.14	4.99	4.57		6.64	4.30	4.74	2.30	1.60	2.06	4.64	1.72
		t ha^−1^	%	mg g^−1^		µg g^−1^		nmol g^−1^		%	µg g^−1^						
2018		9.79	9.39	4.65	70.1	2.53		328.7	9.87	1,075		88.3	1,938	111.7	38.62	11.53	36.66	3.47	2,417
2019		10.30	10.13	4.59	70.9	2.38		374.5	14.92	995		84.7	1,706	262.9	47.11	10.38	26.55	7.86	2,465
2020		8.08	9.40	4.48	71.0	2.60		356.8	10.21	1,055		89.7	1,997	220.0	48.84	10.62	33.88	3.57	2,755
White		7.93	9.09	4.12	71.9	2.50		322.0	1.20	1,125		84.5	1,719	185.8	38.24	9.73	30.77	4.96	2,178
Yellow		9.51	9.84	4.22	70.7	2.63		245.8	19.61	1,134		79.5	1,963	199.3	45.01	11.69	33.90	4.92	2,505
Red		10.73	10.00	5.38	69.4	2.38		492.3	14.20	866		98.8	1,959	209.5	51.33	11.11	32.41	5.01	2,954
BF		9.68	9.48	4.61	70.8	2.51		324.8	11.50	932		87.0	1,941	212.5	58.39	11.36	34.74	4.80	2,524
OF		9.46	9.86	4.65	70.5	2.52		359.6	11.67	1,013		87.5	1,946	220.1	45.13	11.52	33.84	5.89	2,748
Urea		8.97	9.74	4.48	70.4	2.52		379.6	11.54	1,258		88.0	1,855	196.6	40.84	10.45	30.60	5.14	2,504
Con		9.45	9.48	4.56	70.9	2.47		349.4	11.97	964		87.8	1,779	163.7	35.08	10.04	30.26	4.02	2,407

^*^5%, significant at the probability level. SOF, source of variation; df, degrees of freedom; BF, biofertilizer; OF, organic fertilizer; Con, control (no fertilizer); CV, coefficient of variation.

The year did not significantly affect the variation in the starch concentration in the maize grain, and the fertilizer did not significantly affect the variation in the concentrations of the oil, Pphy, phenolics, YP, and DPPH. The genotype effect was insignificant in terms of the variation in the concentrations of Ca and Cu and the year × fertilizer interaction for the variation in the oil and starch concentration. The results indicate that white-kernel maize had a higher starch concentration on average (71.9%), whereas yellow kernel had the greatest average values for Pphy, YP, GSH, Mg, Mn, and Zn (2.63 mg g^−1^, 19.61 µg g^−1^, 1,134 nmol g^−1^, 1,963 µg g^−1^, 11.69 µg g^−1^, and 33.9 µg g^−1^, respectively); the red kernel had the greatest average GY and was also the highest in proteins, oils, phenolics, DPPH, Ca, Fe, Cu, and S (10.73 t ha^−1^, 10.0%, 5.38%, 492.3µg g^−1^, 98.8%, 209.5 µg g^−1^, 51.33 µg g^−1^, 5.01 µg g^−1^, and 2954 µg g^−1^, respectively).

### Correlation between the yield and essential elements

We found differences in the correlation between the GY and the concentration of the essential elements in the kernels (**Table 4**). In general, a significant and negative correlation was observed between the GY and S in the kernels of white and yellow maize (−0.38 and −0.85, respectively). An increase in the GY was followed by a significant increase in the concentration of Mg and Cu in yellow-kernel maize (by 0.56 and 0.57, respectively) and by a significant increase in the concentrations of Mg, Fe, Mn, Zn, and Cu (by 0.53, 0.44, 0.34, 0.53, and 0.66, respectively) in the red maize kernels.

**Table 4:** The correlation between grain yield (GY) and concentrations of the analyzed elements in maize with different kernel colors under different fertilizer treatments [biofertilizer (BF); organic fertilizer (OF); urea; control (Con; no fertilizer)].

Element		Mg	Ca	>Fe	Mn	Zn	Cu	S
Genotype								
White	GY	0.08	−0.23	0.15	0.07	0.14	0.14	−0.38^*^
Yellow		0.56^*^	−0.1	0.26	0.18	−0.22	0.57^*^	−0.85^*^
Red		0.53^*^	0.16	0.44^*^	0.34^*^	0.53^*^	0.66^*^	0.09
Fertilizer treatment								
BF	GY	0.05	0.14	−0.04	0.69^*^	−0.24	0.49^*^	0.55^*^
OF		−0.01	−0.39^*^	0.13	0.19	−0.16	0.47^*^	0.29
Urea		0.08	0.34	0.57^*^	0.48^*^	−0.02	0.19	0.36^*^
Con		0.1	0.29	0.69^*^	0.3	−0.17	0.44^*^	0.15

^*^0.05, significance level.

Across fertilizer treatments, the only significantly negative correlation was observed between GY and Ca in the OF treatment (−0.39). Moreover, a positive correlation was observed between GY and Cu in the OF treatment (0.47), and Mn, Cu, and S were positively correlated with GY in the BF treatment (0.69, 0.49, and 0.55, respectively). In addition, Fe, Mn, and S were positively correlated with GY in the urea treatment (0.57, 0.48, and 0.36, respectively), and Fe and Cu were positively correlated with GY in the control (0.69 and 0.44, respectively).

### Influence of the kernel color and fertilizers on the removal of the essential elements with kernel yield

The removal of mineral elements from the soil with grain yield is an important trait. Fertilizer treatments, genotype, as well as their interaction, expressed a significant impact on the removal of all examined elements with yield (**Table 5**). The average values indicated BF as the treatment with the highest average removal of Mg, Fe, Mn, and Zn (with 10.7%, 41.0%, 13.9%, and 14.7%, respectively, in comparison with control), whereas OF contributed to the greater removal of S (with 13.4% in comparison with control), and urea contributed to the greater removal of Ca and Cu (with 24.9% and 31.6%, respectively, in comparison with control). When the kernel color was considered, red-kernel maize had the highest values of removal for all the examined elements, on average.

**Table 5:** Effect of different fertilizer treatments (biofertilizer (BF); organic fertilizer (OF); urea; control (Con; no fertilizer)) and maize kernel colors on the removal of the analyzed elements with the grain yield (kg ha^-1^) (average of 2018–2020).

	Mg	Ca	Fe	Mn	Zn	Cu	S
White							
BF	12.26 b ± 1.08	1.45 b ± 0.25	0.30 d ± 0.35	0.070 b ± 0.22	0.24 b c ± 0.50	0.036 a b ± 0.13	16.58 a ± 2.43
OF	12.52 b ± 1.07	1.57 b c ± 0.20	0.25 b ± 0.22	0.072 b ± 0.20	0.22 b ± 0.42	0.041 b ± 0.12	18.95 a ± 2.56
Urea	10.99 a ± 1.15	1.05 a ± 0.28	0.22 a ± 0.24	0.061 b ± 0.23	0.18 a ± 0.48	0.033 a b ± 0.15	15.83 b ± 2.90
Con	11.13 a ± 1.04	1.01 a ± 0.18	0.20 a ± 0.20	0.061 b ± 0.20	0.19 a ± 0.42	0.025 a ± 0.09	16.11 a ± 2.49
Yellow							
BF	18.17 e ± 1.31	1.79 b c ± 0.25	0.51 e ± 0.38	0.109 e ± 0.28	0.31 d ± 0.53	0.040 b ± 0.12	23.72 d ± 2.83
OF	15.88 c ± 1.23	1.71 b c ± 0.26	0.35 c ± 0.27	0.099 d ± 0.25	0.28 d ± 0.48	0.046 b ± 0.13	23.05 b c ± 2.77
Urea	14.99 c ± 1.25	1.62 b c ± 0.26	0.32 c ± 0.29	0.086 c ± 0.28	0.25 b c ± 0.52	0.037 a b ± 0.15	20.28 c d ± 3.01
Con	15.22 c ± 1.17	1.39 b ± 0.20	0.30 c ± 0.24	0.088 c ± 0.24	0.26 c ± 0.46	0.037 a b ± 0.12	21.16 c ± 2.69
Red							
BF	18.44 e f ± 1.28	2.09 c ± 0.28	0.58 f ± 0.42	0.107 e ± 0.26	0.31 d ± 0.51	0.044 b ± 0.15	24.76 d ± 2.86
OF	19.36 f ± 1.21	2.07 c ± 0.26	0.52 e ± 0.31	0.111 e ± 0.24	0.32 d ± 0.45	0.057 c ± 0.10	28.09 d ± 2.81
Urea	17.22 d ± 1.28	1.95 c ± 026	0.43 d ± 0.36	0.096 d ± 0.27	0.27 c ± 0.49	0.049 b ± 0.13	24.10 e ± 3.09
Con	17.30 d ± 1.17	1.62 b ± 0.21	0.36 c ± 0.26	0.197 d ± 0.23	0.29 c d ± 0.46	0.036 a b ± 0.12	23.39 d ± 2.62
Mean							
White	11.72 A ± 1.09	1.27 A ± 0.23	0.26 A ± 0.25	0.066 A ± 0.22	0.21 A ± 0.45	0.034 A ± 0.13	16.87 A ± 2.59
Yellow	16.06 B ± 1.24	1.63 A B ± 0.24	0.37 B ± 0.30	0.106 B ± 0.26	0.28 B ± 0.50	0.040 A ± 0.14	22.05 B ± 2.83
Red	18.08 C ± 1.24	1.93 B ± 0.25	0.47 C ± 0.34	0.103 C ± 0.20	0.30 B ± 0.48	0.046 B ± 0.13	25.08 C ± 2.85
BF	16.29 b ± 1.23	1.78 b ± 0.26	0.49 c ± 0.39	0.095 b ± 0.25	0.29 b ± 0.51	0.040 a ± 0.12	21.69 b ± 2.71
OF	15.92 a ± 1.23	1.78 a b ± 0.27	0.37 a b ± 0.30	0.094 a ± 0.26	0.28 a ± 0.50	0.048 a ± 0.15	23.36 a ± 3.00
Urea	14.43 b ± 1.17	1.54 b ± 0.20	0.32 b ± 0.27	0.081 b ± 0.23	0.24 b ± 0.45	0.040 b ± 0.13	20.07 b ± 2.72
Con	14.55 a ± 1.12	1.34 a ± 0.20	0.29 a ± 0.23	0.082 a ± 0.22	0.25 a ± 0.45	0.033 a ± 0.10	20.22 a ± 2.60

Values are presented as mean ± SD. Numbers followed by the same letter do not differ based on the LSD test at p< 0.05.

When the combinations of maize with different kernel colors and fertilizer treatments were considered, the highest removal of Mg, Mn, Zn, Cu, and S was achieved with the yield of red-kernel maize and OF treatment (19.36, 0.11, 0.32, 0.057, and 28.09 kg ha^−1^, respectively). Ca and Fe followed a similar trend, where the highest removal was with the red-kernel maize and BF treatment (2.09 and 0.58 kg ha^−1^, respectively). Following the combination of red-kernel maize and BF/OF, slightly lower values were obtained for the combination of yellow-kernel maize and BF, having values of 18.17, 1.79, 0.51, 0.10, 0.31, and 23.72kg ha^−1^ for Mg, Ca, Fe, Mn, Zn, and S, respectively.

### Influence of the kernel color and fertilizers on the potential bioavailability of the essential elements and DPPH reduction capacity

The potential bioavailability of the metals was reflected through their molar relation with Phy and followed the variations in the concentrations of Phy and the metals in the maize kernels. Notably, all ratios were significantly affected by the genotype, fertilizer type, and their interaction (**Table 6**). Only in the case of Phy/Mg and Phy/Ca ratios were insignificant variations obtained under the influence of fertilizer, and the Phy/Ca and Phy/Cu ratios were insignificant for genotype. Thus, in the white-kernel maize Con treatment, Phy/Mg, Phy/Ca, Phy/Fe, Phy/Mn, Phy/Zn, and Phy/Cu had the lowest values when compared to that in the red-kernel hybrid in BF/OF treatment, which achieved the highest values (to 25.1%, 36.3%, 54.6%, 27.7%, 22.2%, and 42.1%, respectively). Additionally, slightly higher values were obtained in the same treatment (Con) with yellow-kernel maize. Nevertheless, when red-kernel maize was considered, the lowest values for Phy/Ca and Phy/Cu were obtained in the control, whereas reduced values of Phy/Mg, Phy/Fe, Phy/Mn, and Phy/Zn were noted in the urea treatment. Thus, on average, a trend of a reduction in the Phy/metals ratio was observed in the white-kernel maize and control, with the exception of Phy/Zn, which had a lower value in the urea treatment.

**Table 6:** Effect of different fertilizer treatments (biofertilizer (BF); organic fertilizer (OF); urea; control (Con; no fertilizer)) on the molar ratios between phytic acid (Phy) and essential elements, Phy/Mg, Phy/Ca, Phy/Fe, Phy/Mn, Phy/Zn, and Phy/Cu (average for 2018–2020).

	Phy/Mg	Phy/Ca	Phy/Fe	Phy/Mn	Phy/Zn	Phy/Cu
White						
BF	7,276 a b ± 11.28	1,418 b ± 4.51	514 e ± 2.40	94.1 b ± 0.55	387 c d ± 0.97	55.7 b c ± 0.42
OF	7,519 b ± 12.02	1,558 b ± 5.10	352 b c ± 1.69	98.2 b ± 0.59	359 b ± 0.93	64.3 c ± 0.50
Urea	6,999 a b ± 11.15	1,097 a ± 3.58	316 a ± 1.51	88.3 a ± 0.53	316 a ± 0.81	55.3 b c ± 0.43
Con	6,706 a ± 10.79	1,007 a ± 3.32	279 a ± 1.35	83.7 a ± 0.50	310 a ± 0.81	39.6 a ± 0.31
Yellow						
BF	8,282 b c ± 13.68	1,348 b ± 4.55	534 e ± 2.65	112.5 e ± 0.69	385 c d ± 1.02	47.1 a b ± 0.38
OF	7,666 b ± 13.02	1,364 b ± 4.75	390 c ± 1.99	108.4 d ± 0.69	369 c ± 0.94	58.2 b c ± 0.48
Urea	7,794 b c ± 12.86	1,387 b ± 4.69	379 b c ± 1.88	101.3 c ± 0.62	354 b c ± 0.90	50.8 b ± 0.41
Con	7,251 a ± 12.14	1,088 a ± 3.73	330 b ± 1.66	94.9 b ± 0.59	331 a b ± 0.97	46.6 a b ± 0.38
Red						
BF	8,484 c ± 13.89	1,581 b ± 5.11	615 f ± 2.92	111.2 e ± 0.66	386 c d ± 0.96	53.0 b ± 0.41
OF	8,955 c ± 13.37	1,576 b ± 4.82	550 e ± 2.47	115.7 e ± 0.65	399 d ± 0.87	68.4 c ± 0.49
Urea	8,137 b c ± 12.60	1,522 b ± 4.83	466 d ± 2.17	102.1 c ± 0.59	346 b ± 0.89	60.2 b ± 0.45
Con	8,551 c ± 12.12	1,321 b ± 3.86	413 c ± 1.77	108.4 d ± 0.57	388 c d ± 0.93	46.1 a b ± 0.32
Mean						
White	7,125 A ± 11.31	1,270 n.s. ± 4.13	365 A ± 1.74	91.1 A ± 0.54	343 A ± 0.88	53.7 n.s. ± 0.41
Yellow	7,749 A B ± 12.91	1,297 n.s. ± 4.43	408 A ± 2.05	104.3 B ± 0.65	360 A ± 0.98	50.7 n.s. ± 0.41
Red	8,532 B ± 12.89	1,500 n.s. ± 4.66	511 B ± 2.33	109.3 B ± 0.62	380 B ± 0.90	56.9 n.s. ± 0.42
BF	8,014 n.s. ± 12.77	1,449 n.s. ± 4.72	554 d ± 2.65	105.9 b ± 0.63	386 b ± 0.99	52.0 a b ± 0.40
OF	8,047 n.s. ± 12.80	1,499 n.s. ± 4.89	431 c ± 2.05	107.4 b ± 0.64	375 b ± 0.97	63.6 b ± 0.49
Urea	7,644 n.s. ± 12.21	1,335 n.s. ± 4.37	387 b ± 1.86	97.2 a ± 0.58	339 a ± 0.87	55.4 b ± 0.43
Con	7,503 n.s. ± 11.70	1,139 n.s. ± 3-64	341 a ± 1.59	95.6 a ± 0.56	343 a ± 0.86	44.1 a ± 0.33

Values are presented as mean ± SD. Numbers followed by the same letter do not differ based on the LSD test at p < 0.05; n.s., not significant.

The results from **Table 7** pointed to the presence of a significant and positive correlation between the reduction capacities of the DPPH radical, Pphy, and GSH in the white- (0.68 and 0.44, respectively) and yellow-kernel maize (0.74 and 0.38, respectively), whereas there was a negative correlation with YP (−0.74 in hybrid with yellow kernels). In the red-kernel maize, there was a significant and positive correlation between the DPPH, phenolics, and GSH (0.46 and 0.55, respectively). When the fertilizer treatments were considered, a positive correlation between the DPPH and phenolics was observed in all treatments. Furthermore, there was a significant and negative correlation between the DPPH, Pphy, and GSH in the OF (−0.64 and −0.44, respectively) and Con (−0.57 and −0.70, respectively) treatments, and there was only a significantly negative correlation with GSH in the urea treatment (−0.41).

**Table 7:** The correlation between the reduction capacity of the DPPH radical, concentration of the analyzed antioxidants in maize with different kernel colors, and the application of different fertilizers (biofertilizer (BF); organic fertilizer (OF); urea; control (Con; no fertilizer)).

Antioxidant		Pphy	Phenolics	GSH	YP
Genotype					
White	DPPH	0.68^*^	0.04	0.44^*^	−0.13
Yellow		0.74^*^	−0.14	0.38^*^	−0.74^*^
Red		−0.09	0.46^*^	0.55^*^	0.1
Fertilizer treatment					
BF	DPPH	−0.28	0.89^*^	−0.36	−0.2
OF		−0.64^*^	0.85^*^	−0.44^*^	0.09
Urea		−0.27	0.84^*^	−0.41^*^	−0.09
Con		−0.57^*^	0.78^*^	−0.70^*^	−0.08

^*^0.05, significance level.

### Interdependence between the kernel color, fertilizer treatments, and GY and the chemical composition

The PCA, as a dimension reduction method, indicated that the first axis explained 48.1% of the total variability, the second axis explained 25.0%, the third axis explained 10.5%, and the fourth axis explained 7.6%. The GY and protein, oil, YP, Mg, Ca, Fe, Mn, Zn, and Cu concentrations correlated significantly and positively with the first axis, whereas starch was negatively correlated. Furthermore, a significant and positive correlation was found between the second axis and Pphy, whereas it was negatively correlated with the phenolics and DPPH reduction capacity. Only Cu was significantly positively correlated with the third axis.

**Figure 1:**
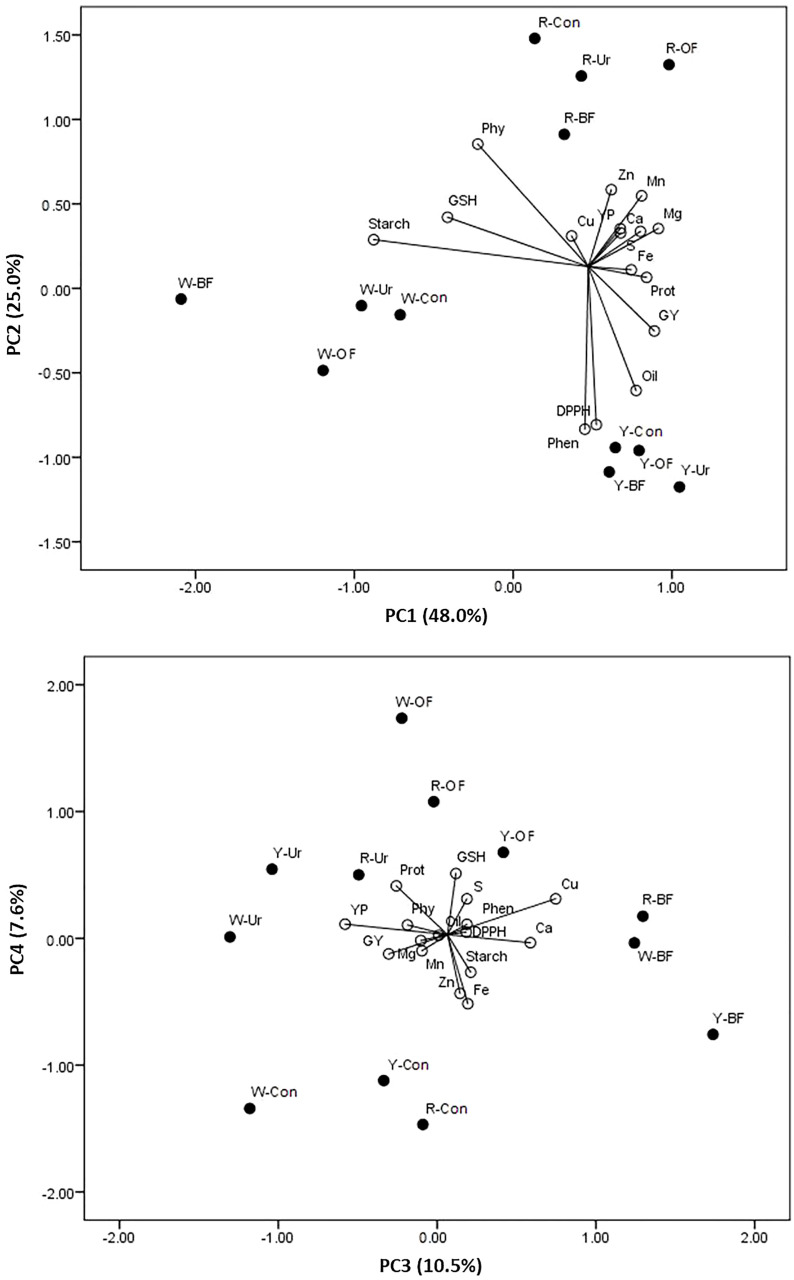
Principal component analysis of the grain yield (GY), protein (Prot), oil, starch, phytic phosphorus (Phy), phenolics (Phen), yellow pigment (YP), and glutathione (GSH) contents, reduction capacity of the DPPH radical (DPPH), and concentrations of Mg, Ca, Fe, Mn, Zn, Cu, and S in maize with different kernel colors (W, white; Y, yellow; R, red), under different fertilizer treatments(BF, biofertilizer; OF, organic fertilizer; Ur, urea; Con, control).

Considering the mutual impact of the kernel color and fertilizer treatments on the variability of each trait, it is notable that the highest variability in the starch concentration and GSH was in the white-kernel maize, mainly in the urea and control treatments and to a lesser degree in the BF and OF treatments (**Figure 1**). The starch concentration in the yellow- and red-kernel hybrids in the control and in the GSH in the OF treatment also showed slight variability. Greater variability in the GY, oil, phenolics, and reduction capacity of the DPPH radical was observed in the yellow-kernel maize in all fertilizer treatments. Moreover, the GY variability was slightly affected by the urea treatment for all three kernel colors. Variability in the Pphy concentration was mainly caused by the BF and urea treatments, and a slight variation was caused by no fertilization (Con) in the red-kernel hybrid. Greater variability occurred in the YP, Zn, Mn, Mg, Ca, and S in the red kernel and OF combination.

## Discussion

The maize kernel plays an important role in the human diet in many regions globally. Genotypes with various kernel colorations, ranging from intense yellow to red, purple, or even blue and black, are very popular (Žilić et al., 2012; Sha et al., 2016; Suriano et al., 2021); however, in some regions, the white kernel is mainly used for human nutrition. Owing to a lack of information on the status of the other important nutrients, such as essential elements, this study provides valuable information on the ability to enhance the concentrations of essential elements and also improve their potential bioavailability from kernels of differently colored maize, aided by fertilization.

### Year as a source of variation

As the experiment was performed under dry farming conditions, we demonstrated that the year and its interaction with other factors, such as the genotype, had the greatest impact on the variability of kernel characteristics. It is well known that meteorological variations, especially drought, are of great importance for maize yield as well as protein storage, including the absorption and accumulation of mineral elements from the soil (Ben Mariem et al., 2021). Dry conditions could severely affect crop growth and kernel filling and, thus, yield potential; however, they could also have a positive impact on the nutritional quality by increasing the protein level and accumulation of some antioxidants and mineral elements in the kernels (Saini and Keum, 2018). Even phytate, as a genotype characteristic, varied significantly across the years, confirming that climate could affect its concentration in the cereal grain (Perera et al., 2018).

### Variability in the yield and chemical composition based on the kernel color

With respect to grain yield and macronutrients, it appears that red-kernel maize has greater yield potential and could be considered a good source of protein and oil and, therefore, should be a valuable part of the human diet (Sha et al., 2016). Considering the other two hybrids, white-kernel maize could be a good source of starch. It is also high in phenolics and GSH but low in essential nutrients, making it a good source of antioxidants but not minerals.

When comparing the kernels of different colors, it was obvious that the yellow kernel was richer in yellow pigment, as was expected, and in phytate and GSH, as important antioxidants, which supported the positive increasing trend of the scavenging capacity of the DPPH radical. This implies that maize is a good source of various antioxidants (Žilić et al., 2012; Sha et al., 2016; Suriano et al., 2021); our study indicates that the yellow kernel shows favorable characteristics in this regard. Additionally, the yellow-kernel maize was also high in the essential elements Mg, Mn, and Zn. However, slightly lower values of removal were found for this hybrid, particularly in Mg and Cu, which were positively correlated with the grain yield increase. In contrast to the metals, only S was negatively correlated with the grain yield increase in the white- and yellow-kernel hybrids. However, the red kernel was superior with respect to phenolics and scavenging capacity of the DPPH radical, as well as a high concentration of GSH, emphasizing a greater phenolic level in terms of the antioxidant activity of the maize kernel, when compared to other antioxidants (Žilić et al., 2012; Das and Singh, 2016). The same genotype was also high in Ca, Fe, Cu, and S. Thus, these differences again provided evidence that nutrient remobilization from the vegetative parts into the grain is highly dependent on the genotype (Ray et al., 2020) and could be the main reason for the highest removal of mineral nutrients with yield. This finding was additionally supported by the significantly positive correlation of the grain yield and all the examined essential elements, except for Ca and S. Interestingly, despite having the highest S level, the red-kernel maize had the lowest concentration of thiolic protein, GSH. Nevertheless, the white-kernel maize was still relatively high in the GSH, phenolics, phytic P, and the scavenging capacity of the DPPH radical, which was positively linked with the increasing level of phytic P and GSH.

Greater accumulation of the essential elements in the maize kernels does not necessarily indicate greater accessibility for humans and monogastric animals, which is mainly dependent on the concentrations of the various antinutrients, such as phytic acid, in the grain (Iwai et al., 2012; Brouns, 2021; Feizollahi et al., 2021). Thus, it is important to know the molar ratio between phytic acid and the essential metals as an indicator of their potential bioavailability (Johnson et al., 2013; Wang et al., 2015). Even though the red-kernel maize had the lowest average phytic acid concentration, the value of the phytic acid/essential metals ratio was the greatest in its kernels. Considering that a high level of phenolics could interfere with the accessibility of essential metals (Johnson et al., 2013), it can be assumed that the potential bioavailability, mainly of Ca, Fe, Cu, and other elements, from the red-kernel maize, was compromised, weakening its potential as a highly accessible source of essential elements. Nevertheless, fertilization, such as with urea and organic fertilizer, significantly reduced the ratio of phytic acid and essential elements in the kernel of this genotype, implying that fertilization practices could be successfully used to enhance the chemical composition of desirable traits in the kernels. Compared to the red-kernel maize, the yellow-kernel hybrid had a slightly lower phytic acid/essential metals ratio and the lowest phytic acid/Cu ratio, in combination with the greatest values for the promoters, yellow pigment, and GSH (which enhance the bioavailability of the essential metals), which could emphasize the yellow hybrid as a highly accessible source of Cu and potentially Mg, Mn, and Zn as well. Some elements are considerably lacking in diets worldwide (Lowe, 2021). When compared to the red- and yellow-kernel hybrids, the white-kernel hybrid had the lowest phytic acid/essential metals ratio, making it a desirable choice for highly available essential elements.

### Variability in the yield and chemical composition, governed by fertilizer type

Fertilization is an important practice to optimize crop growth, fitness, and yield potential, as well as boost the synthesis and accumulation of important nutrients in the edible parts of plants, such as the grain in maize, thus improving their nutritional quality. Until now, fertilization has mainly considered the application of macronutrients, such as N, P, and K. Meanwhile, the rising trend in soil devastation and the increasing requirements for the production of nutrient-dense crops (FAO, 2022) necessitate sustainable strategies that will increase efficiency and faster absorption of nutrients. Both organic and biofertilizers are used to sustain/improve soil fertility and uphold crop growth through improved nutrient absorption efficiency. They also overlap in benefits in terms of increasing the diversity and number of beneficial soil microbiota (Du et al., 2022).

The findings of this study showed that biofertilizer had a positive impact on the average grain yield as well as Fe and Zn accumulation in the maize grain, demonstrating that by promoting the activity of soil microbiota, the absorption of essential elements, increased crop fitness, and grain yield could be realized. Consequently, biofertilizer contributed to the greater removal of essential elements with yield, mainly with the red-kernel hybrid, such as Ca and Fe, whereas organic fertilizer was effective for Mg, Mn, Zn, Cu, and S removal. Similar findings were reported for sweet maize, which was grown after cover crops and biofertilizer, and dent maize, which was intercropped with soybean and biofertilizer (Dragicevic et al., 2015; Dragicevic et al., 2021). Notably, the incorporation of Zn fertilizers into the soil can enhance microbial metabolism, positively affecting Zn absorption, whole-plant metabolism, and promoting further pollen viability and kernel number, thus increasing the yield; however, this effect is highly dependent on the genotype (Liu et al., 2020; Xiao et al., 2022). This could explain the highest grain yield achieved by the red-kernel hybrid in biofertilizer treatment. Although organic fertilizer was important to increase macronutrient accumulation in the maize kernels (oils and proteins), it also enhanced the absorption and accumulation efficiency of essential elements, such as Mg, Ca, Mn, Cu, and S. Nevertheless, the findings revealed that urea is essential for the antioxidant status of maize kernels, as it improved the scavenging capacity of the DPPH radical and increased the accumulation of Pphy, phenolics, and GSH. The phenolics were positively correlated with the scavenging capacity of the DPPH radical in all the treatments, confirming their importance in the antioxidant response. It is well known that urea promotes the absorption and accumulation of Zn and Fe in the grains of various crops (Yuan et al., 2017; Pal et al., 2021). In this study, urea parallel increased the grain yield and Fe, Mn, and S in the maize grain.

The phytic P concentrations in the treatments with organic fertilizer and urea were very similar, indicating that P, as well as N, can play an important role in phytic acid accumulation (Ning et al., 2009; Kaplan et al., 2019). Even though phytic acid is an important antioxidant and, thus, can considerably increase the antioxidant potential of plants (Akin-Idowu et al., 2017; Pramitha et al., 2021), in this study, it negatively correlated with the scavenging capacity of the DPPH radical in the organic fertilizer and control treatments. This indicated that apart from the genotype, other cropping practices could influence the share of phytic acid, affecting antioxidant activity. It is well known that urea is successfully used for biofortification to enhance Zn and Fe accumulation in crop grains (Pal et al., 2021; Kaur and Singh, 2022). In this study, urea primarily decreased the ratio of phytic acid with Mg, Fe, Mn, and Zn even in red-kernel maize, thereby contributing to their better potential accessibility.

The study limitations are attributed to the fact that only one soil type, the chernozem soil type, was considered, and the inclusion of soils lacking in multiple elements could more extensively explain the potential impact of applied fertilizers. From the viewpoint of potential bioavailability, further research comprising experiments *in vitro* and *in vivo* could provide a new avenue for research and integrate results from agricultural and nutritional/medical sciences regarding the nutritional value of variously colored maize kernels with elevated concentrations of essential elements under real-time conditions.

## Conclusion

The importance of maize as a staple crop and a source of various nutrients was supported by this study. The contribution of maize was determined by comparing the yield and chemical composition of differently colored kernels, with a focus on different fertilizer types as a possible tool for agronomic biofortification.

When the hybrids with differently colored kernels were compared, the white kernel was the best in terms of variability in the starch and GSH concentrations, while the yellow-kernel hybrid had a greater potential for achieving a high grain yield, oil and phenolic concentrations, and greater scavenging capacity of the DPPH radical. The red-kernel hybrid had the highest potential to enhance the kernel composition, based on greater variability in all the examined essential elements and yellow pigment, and there was a greater potential for reducing the phytic acid concentration, which could lead to an increase in its potential bioavailability. Thus, the impact of the genotype on the variability in the examined traits was significant.

The fertilizer type, such as bio- and organic fertilizers, also played an important role in improving kernel quality with respect to the accumulation of essential elements and their greater removal with yield. From such viewpoint, biofertilizer was beneficial for grain yield as well as greater accumulation of proteins, Fe, Cu, and S and antioxidants status, particularly when red-kernel hybrid was considered, while organic fertilizer was mainly efficient for greater accumulation of macronutrients in the kernels, too, including essential elements, such as Mg, Ca, Mn, Cu, and S. Although urea is a less sustainable fertilizer, it was important in enhancing the antioxidant status and increasing the potential Zn bioavailability from the maize kernels.

The results of this study can be used to determine an appropriate genotype based on the antioxidants and/or essential elements targeted for kernel enhancement. We recommend that, in general, all three genotypes should be included in human diets in a cyclical manner and that the share of maize products, as a rich source of phytonutrients, should be increased.

## Data availability statement

The original contributions presented in the study are included in the article. Further inquiries can be directed to the corresponding author.

## Author contributions

VD and MSi contributed to the experiment design. VD and MSt conducted the chemical analysis. VD, MB, and PK conducted the statistical analyses. VD, MB, and Msi wrote the manuscript. MB, Msi, and MT organized the experiment. PK, Msi, and IT edited the manuscript. Msi initiated the experiment. Msi and MT acquired equipment and funding. All the authors contributed to the revision of the manuscript and read and approved the final version.

## Funding

This research was supported by the Ministry of Education, Science and Technological Development, Republic of Serbia, under Grant no. 451-03-68/2022-14/200040.

## Acknowledgments

The authors are grateful to Branka Radovanović, Biljana Noro, Milan Kostić, and Miroslav Maksimović for their effort and dedication in conducting the experiment.

## Conflict of interest

The authors declare that the research was conducted in the absence of any commercial or financial relationships that could be construed as a potential conflict of interest.

## Publisher’s note

All claims expressed in this article are solely those of the authors and do not necessarily represent those of their affiliated organizations, or those of the publisher, the editors and the reviewers. Any product that may be evaluated in this article, or claim that may be made by its manufacturer, is not guaranteed or endorsed by the publisher.
